# Purple Tea and Its Extract Suppress Diet-induced Fat Accumulation in Mice and Human Subjects by Inhibiting Fat Absorption and Enhancing Hepatic Carnitine Palmitoyltransferase Expression

**Published:** 2015-06

**Authors:** Hiroshi Shimoda, Shoketsu Hitoe, Seikou Nakamura, Hisashi Matsuda

**Affiliations:** 1 Research & Development Division, Oryza Oil & Fat Chemical Co., Ltd., 1 Numata, Kitagata-cho, Ichinomiya, Aichi 493-8001, Japan;; 2 Kyoto Pharmaceutical University, 1 Shichono-cho, Misasagi, Yamashina-ku, Kyoto 607-8412, Japan

**Keywords:** 1,2-di-*O*-galloyl-4,6-*O*-(*S*)-hexahydroxydiphenoyl-*β*-D-glucose, caffeine, carnitine palmitoyltransferase, epigallocatechin gallate, purple teac

## Abstract

A number of clinical trials have been completed using green tea and black tea to investigate their effect in controlling weight in overweight adults. The results of these investigations, however, have often been contradictory, with some trials reporting positive effects of tea supplementation and some trials reporting no effect. As a result, the use of these teas for weight loss is controversial. Purple tea is a variety of green tea developed in Kenya (called TRFK306), which in addition to certain tea constituents found in green tea, also contains anthocyanins. The major constituents in the leaves of purple tea are caffeine, theobromine, epigallocatechin (ECG), epigallocatechin gallate (EGCG) and 1,2-di-*O*-galloyl-4,6-*O*-(*S*)-hexahydroxydiphenoyl-*β*-D-glucose (GHG). We investigated the efficacy of purple tea extract (PTE) on diet-induced fat accumulation in mice. PTE administration (200 mg/kg) significantly suppressed body weight gain, liver weight, abdominal fat and triglycerides in serum and liver. Protein expression of carnitine palmitoyltransferase (CPT) 1A was also enhanced. In olive oil loaded mice, PTE (100 mg/kg) and caffeine (25 mg/kg) suppressed fat absorption. PTE (10 μg/mL) and GHG (10 μg/mL) also enhanced protein expression of CPT1A in HepG2 hepatoma. Moreover, 4-week daily consumption of purple tea drink in humans improved obesity parameters compared to baseline, including body weight (79.9 ± 3.1 kg vs 80.8 ± 3.2, *p*<0.05), body mass index (BMI) (26.8 ± 0.6 vs 27.0 ± 0.6, *p*<0.05) and body fat mass (21.0 ± 1.4 kg vs 21.8 ± 1.5, *p*<0.01). In conclusion, PTE could control diet-induced weight gain by suppression of fat absorption and enhancement of hepatic fat metabolism

## INTRODUCTION

The prevalence of obesity continues to increase and be a major health issue in the US and in Latin American countries (1, 2). A number of weight-loss supplements containing a variety of ingredients have been used in those countries (3). Green tea (the leaves of *Camellia sinensis*) and green tea preparations, have been marketed and consumed as a supplement for weight loss and weight management for a number of years. Reviews of the clinical evidence, however, have reported both positive effects (4, 5) and no effects (6, 7) on weight loss.

The mechanisms of action for green tea’s potential role as a weight loss ingredient appear to involve a combination of catechins and caffeine. Catechins upregulate hepatic lipid-metabolizing enzymes, including carnitine palmitoyltransferase (CPT) 1 to stimulate fat oxidation (8). They also inhibit catechol-*O*-methyltransferase (COMT) activity and increase norepinephrine and adenyl cyclase. Consequently glucose uptake is decreased and lipolysis is enhanced. On the other hand, caffeine stimulates sympathetic nervous system, hormone sensitive lipase and up-regulation of uncoupling proteins (UCPs), leading to energy expenditure and fat oxidation (8). Oolong tea, a deeply fermented green tea also has been shown to have anti-obesity effects (9), as *in vitro* studies suggest that polymerized catechin derivatives inhibit intestinal lipase activity and reduce fat absorption from gut (10, 11).

**Figure 1:**
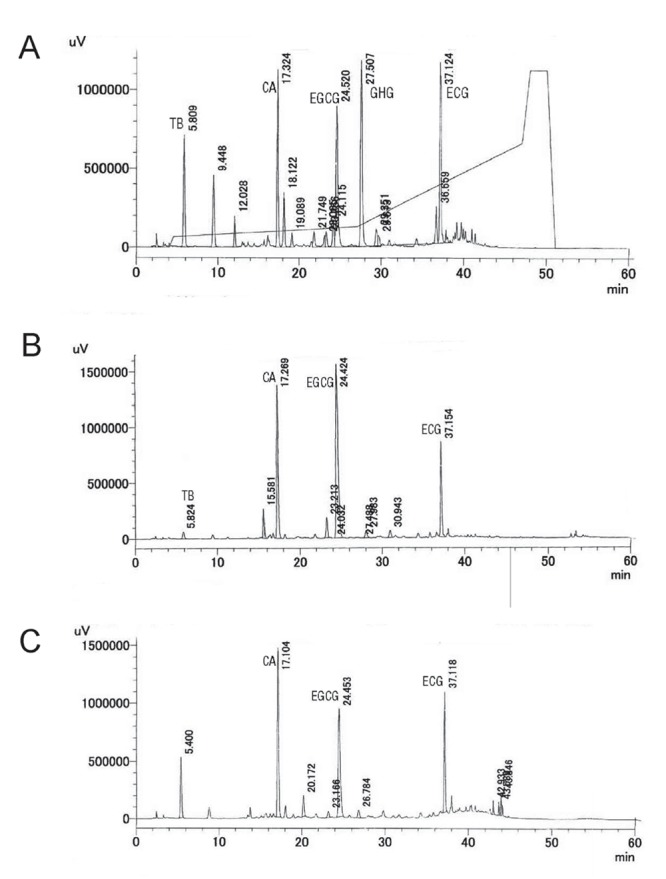
HPLC Chromatogram of 50% Ethanolic Extract of Tea Leaves. Purple tea (A), Japanese green tea (B) and Indian black tea (C) were extracted with 50% ethanol at 40ºC for 2 hr and the solvents were evaporated. HPLC chromatogram was recorded at 280 nm by UV detector. HPLC condition was described in the materials and methods. TB, theobromine; CA, caffeine; EGCG, epigallocatecin gallate; GHG, 1,2-di-*O*-galloyl-4,6-*O*-(*S*)-hexahydroxydiphenoyl-*β*-D-glucose; ECG, epicatechin gallate.

Purple tea is a variety of *Camellia sinensis* developed by the Tea Research Foundation of Kenya (TRFK) and is currently cultivated in Kenya (12). Purple tea leaves are processed by the same method used to process green tea. In addition to the usual polyphenolic compounds found in green tea, such as epigallocatechin gallate (EGCG) and epicatechin gallate (ECG), purple tea is uniqe in that it also contains anthocyanidins (malvidin, peralgonodin and cyanidin 3-*O*-galactoside) and 1,2-di-*O*-galloyl-4,6-*O*-(*S*)-hexahydroxydiphenoyl-*β*-D-glucose (GHG), a hydrolysable tannin (12). Purple tea also differs from other varieties of *Camellia sinensis* in its caffeine content, which is relatively lower in comparison to green and black tea (13, 14). Hence, the HPLC chromatogram of purple tea differs from green tea and black tea (Fig. 1). The reported biological activities of purple tea include anti-trypanosome (15) and cerebral antioxidant (16) activities. However, anti-obesity effects have yet to be evaluated. Hence, we prepared purple tea extract (PTE) and evaluated the effect on diet-induced fat accumulation in mice. In addition, the anti-obesity mechanism and active compounds were investigated. Furthermore, a small-scale clinical trial was conducted in mildly obese human subjects.

## MATERIALS AND METHODS

### Preparation of PTE and Isolation of the Constituents in PTE

For the biological assay, PTE was prepared from dried leaves of purple tea (*Camellia sinensis*, TRFK306) cultivated in 2012 in the foothills of Mt. Kenya, including Thika and Kerugoya areas. Dried leaves (1 kg) were extracted with 50% w/w aqueous ethanol (10 L) for 2 h at 40°C. The solvent was evaporated to obtain PTE (338 g). The principal constituents in PTE were theobromine (1.6%), caffeine (4.4%), EGCG (9.8%), GHG (7.4%) and ECG (5.8%).

To isolate these constituents, PTE (100 g) was dissolved in H2O (1 L) and extracted with AcOEt (2 L) 3 times to obtain AcOEt fraction (35.7 g). Then H_2_O layer was extracted with *n*-BuOH (1.5 L) to obtain BuOH fraction (32.4 g). BuOH fraction (25 g) was separated by ODS (375 g) column by 30 to 100% MeOH. The fraction (15 g) extracted with 30% MeOH was loaded onto DiaionTM HP21 column (150 g, Mitsubishi Chemical, Tokyo, Japan) and washed with 10% MeOH. Then 30% MeOH was loaded to obtain 30% MeOH fraction (8.2 g). The 30% MeOH fraction (4.3 g) was separated by HPLC equipped with ODS column (Chromatorex ODS, Φ20 × 250 mm, Fuji Silysia Chemical Ltd., Kasugai, Japan) using 30% MeOH to obtain fraction 1 to 7. Refractive index (RI) detector was used for detection. Fraction 2 was purified by HPLC column (Develosil^TM^ ODS-SR-5, Φ20 × 250 mm, Nomura Chemical Co. Ltd., Seto, Japan) using 40% MeOH to obtain caffeine (117 mg). Fraction 3 and 4 were mixed and purified by HPLC column (Develosil^TM^ ODS-SR-5) using 40% MeOH to obtain theobromine (100 mg). Fraction 6 was purified by HPLC column (Develosil^TM^ ODS-SR-5) using 40% MeOH to obtain GHG (174 mg) and ECG (45 mg). Isolated compounds, including GHG (12), were identified by comparison of ^13^C- and 1H-NMR spectra and mass spectra with reported values.

### HPLC Determination of the Principal Constituents in PTE

For evaluation of seasonal changes of the principal constituents in PTE, the leaves harvested from January to September in 2013 in the previously described areas were used. PTE prepared from the leaves was analyzed by HPLC using the following conditions: TSKGEL ODS-80TS QA (Φ4.6 mm × 150 mm, Tosoh, Tokyo, Japan) and UV detector 280 nm); flow rate was fixed at 0.7 mL/min and 5% acetonitrile containing 0.1% trifluoro acetic acid (TFA) and acetonitrile were used for solvents A and B, respectively. Column temperature was set at 35°C. Gradient conditions were 0 to 4 min (solvent A: 100%), 4 to 4.5 min (solvent A: 100 to 95%), 4.5 to 27 min (solvent A: 95 to 90%), 27 to 47 min (solvent A: 90 to 50%), 47 to 48 min (solvent A: 50 to 15%), 48 to 50 min (solvent A: 15%).

### Animals and Cells

The experiments were performed in accordance with the Guidelines for the Proper Conduct of Animal Experiments (Special Council of Japan, June 1, 2006). Male ICR mice aged 5 and 10 weeks old were purchased from Japan SLC Co. Ltd. (Hamamatsu, Japan) and preliminary acclimated for a week in a humidified room (50 ± 5%) with 22 ± 2°C.

HepG2 hepatoma were obtained from JCRB cell bank (Sennan, Japan).

### Reagents

Triglyceride E Test Wako, clofibrate, skimmed milk, olive oil, Dulbecco modified Eagle medium (D-MEM) and fetal calf serum (FCS) were purchased from Wako Pure Chemical Industries Ltd. (Osaka, Japan). Laemmli sample buffer and polyvinylidene fluoride (PVDF) membrane were obtained from Bio-Rad Laboratories Inc. (Collage Station, AZ, USA). RIPA lysis and extraction buffer, protease and phosphatase inhibitor cocktail and Pierce Western Blotting Substrate Plus were purchased from Thermo Fischer Scientific Inc. (Waltham, MA, USA). Mouse anti-CPT1A antibody was obtained from Abcam (Cambridge, UK). Horse radish peroxidase (HRP)-conjugated goat anti-mouse IgG was purchased from Merck Millipore (Darmstadt, Germany). Anti-β-actin monoclonal antibody was obtained from Sigma-Aldrich (St. Louis, MO, USA). Orlistat (Xenical) was obtained from F. Hoffmann-La Roche, Ltd. (Basel, Switzerland).

### High Fat Diet-Induced Body Weight and Lipid Parameter Changes in Mice

As described in our previous study, the treatment and control groups of mice (11 weeks old) were fed high-fat diet 32 (HFD, Clea Japan Inc., Tokyo, Japan) for 17 days (17). For normal mice group, Clea Rodent Diet CE-2 (Clea Japan) was fed as the standard diet. PTE (200 mg/kg) was administered orally to treatment group once a day for 17 days. All groups were anesthetized with isoflurane and blood was collected from abdominal ventral aorta and triglyceride content (TG) was measured with Triglyceride E Test Wako. The liver, epidydimal and perirenal fats were removed and their weights were measured. After weighing, the liver specimen was stored at -80°C for evaluation of TG content and CPT1A protein expression. To determine TG, the specimen (approx. 50 mg) was homogenized in the mixture of chloroform and MeOH (2:1) and centrifuged (3,000 r.p.m., 10 min) to obtain the supernatant. The solvent was evaporated by N2 gas and H2O (1 mL) was added to disperse the homogenate. The TG content was measured by Triglyceride E Test Wako. To determine the CPT1A expression, the liver specimen was homogenized in RIPA extraction and isolation buffer containing protease and phosphatase inhibitor cocktail. The protein concentration of homogenate was adjusted with RIPA buffer to 1 mg/mL. The lysate was mixed with the same volume of Laemmli sample buffer [62.5 mM Tris-HCl, 2% SDS, 5% 2-mercaptoethanol, 25% glycerol and 0.01% bromophenol blue] and heated at 95ºC for 5 min. The heated solution (15 μL) was electrophoresed on 10% sodium dodecyl sulfate (SDS) gel. Separated protein was then transferred to a PVDF membrane. After blocking of the membrane with 5% skimmed milk, CPT1A expression was detected by mouse anti-CPT1A antibody (1:5,000) and HRP-conjugated anti-mouse IgG (1:25,000). β-actin was detected by anti-β-actin antibody (1:20,000) and HRP-conjugated anti-mouse IgG (1:25,000). Detection was performed using by Pierce Western Blotting Substrate Plus and an imaging system (ImageQuant LAS500, GE Health Care, Fairfield, CT, USA).

### Serum Triglyceride Changes in Olive Oil-loaded Mice

The experiment followed the method described in our previous report (18). Mice (6 weeks old) were fasted for 12 hr and blood samples were collected from orbital sinus under anesthesia by glass capillary. Thirty minutes later, PTE or its constituents were given orally to the mice. Then, olive oil (5 mL/kg) was orally loaded 1 hr later and blood samples were collected 2, 4 and 6 hr later. The blood samples were centrifuged (3,000 r.p.m, 5 min) to obtain serum. Triglyceride contents were measured by Triglyceride E Test Wako.

### CPT1A Protein Expression in HepG2 Liver Hepatoma

HepG2 cells (4 × 10^4^ cells) suspended in 500 μL of D-MEM containing 10% FCS were seeded onto a 24-well culture plate and incubated at 37°C under 5% CO_2_ atmosphere for a day. The solution of PTE or its constituents was added to cells and cultured for another day. Cells were collected by 200 μL of RIPA extraction and isolation buffer containing protease and phosphatase inhibitor cocktail. The expression of CPT1A and β-actin were determined by previously described procedure.

### Evaluation of Obesity Parameter Changes in Human Subjects by Ingestion of Purple Tea

The experiment was performed in accordance with the 6^th^ revision of the Declaration of Helsinki of 2008. As test subjects, 10 males aged 32 to 69 (averaged age: 47.1 years old) with mild obesity (BMI>24) were chosen. Exclusion criteria included: daily intake of purple tea; allergic reactions; and serious disorders such as diabetes, liver disorders, kidney disorders or cardiovascular disease. Before starting the test, a written IRB-approved explanation and agreement was provided to the test subjects. A thorough explanation on the test was followed by a questions and answers session, and confirmation that they thoroughly understood the details of the test. Test subjects then submitted written agreement of their consent.

On the first day of the test period, blood samples were collected from fasted test subjects. BMI, bodyweight, body fat mass, abdominal fat, muscle amount, body fat ratio, muscle ratio, basal metabolism, moisture, waist, hip, waist:hip ratio, abdominal subcutaneous fat thickness, right upper arm subcutaneous fat thickness, were measured. After the measurement, dried purple tea leaves (1.5 g/portion) were given to each subject. The subjects ingested the tea extracted from the purple tea portion with hot water (100-200 mL) twice a day for 4 weeks. On the last day of the test period, measurements of obesity parameters were carried out again to compare them with the values before ingestion.

### Statistical analysis

Data are presented as mean ± standard error (SE). For statistical comparisons in the cell experiment, one-way analysis of variance (ANOVA) followed by Dunnett’s test was performed. Paired *t*-test was used for human studies. A value of *p*<0.05 was considered to indicate statistical significance.

## RESULTS

### Seasonal Changes of Major Constituents in PTE

**Figure 2:**
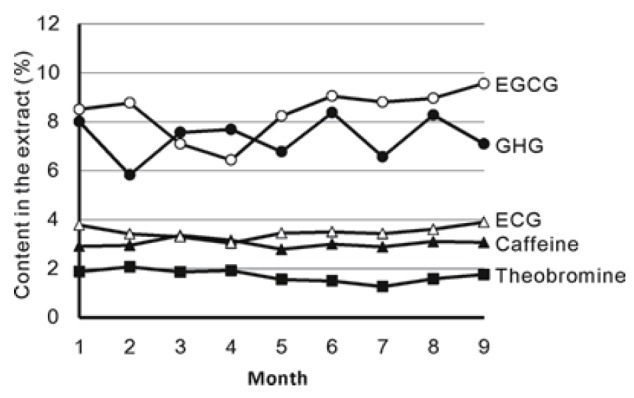
Seasonal Changes of Catechins, GHG and Caffeine in PTE.

The contents of caffeine and theobromine were relatively stable from January to September (Fig. 2). The range of caffeine and theobromine contents were 2.7 to 3.4% and 1.2 to 2.1%, respectively. Among polyphenolic compounds, ECG content was relatively stable, ranging from 3.0 to 3.9%. EGCG content decreased from February to April and then increased in June. The maximum content of EGCG reached to 9.6% in September. GHG content varied between 6.2 to 8.4%.

### Effects of PTE on High Fat Diet-induced Fat Accumulation in Mice

**Figure 3:**
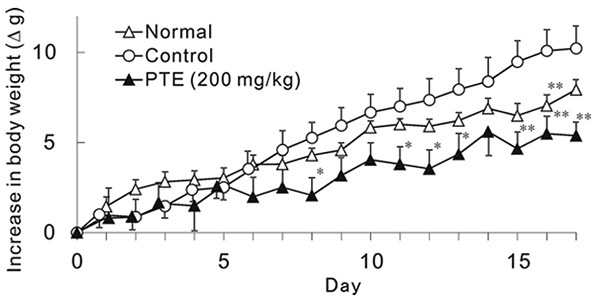
Effect of PTE on HFD-induced Bodyweight Gain in Mice. Each point represents mean with the S.E. (n=5-6). Asterisks denote significant differences from control at ^*^ *p*<0.05, ^**^ *p*<0.01, respectively. PTE (200 mg/kg) was given once a day for 17 days.

The body weights of the control group exceeded those of normal group from day 7 to12 (Fig. 3). PTE (200 mg/kg) suppressed bodyweight gain from day 6 to 12 compared to control group. Final bodyweight in PTE group was significantly suppressed and the average body weight was same as normal group (Table 1). The weights of liver, epididymal fat and perirenal fat were also significantly suppressed by PTE. Regarding triglyceride content, serum TG and hepatic TG were also suppressed by PTE.

**Table 1:** Effect of PTE on Bodyweights, Liver and Epidydimal Fat Weights in Mice Fed HFD

	Dose (mg/kg)	Body weight (g)	Liver (g)	Epididymal fat (g)	Perirenal fat (g)	Serum TG (mg/dL)	Liver TG (μg/mg)

Normal	—	40.8 ± 0.8^a^	1.71 ± 0.04	0.73 ± 0.06^b^	0.28 ± 0.03^b^	124 ± 12	12.9 ± 0.9
Control	—	45.8 ± 1.5	1.77 ± 0.05	1.94 ± 0.14	0.76 ± 0.08	121 ± 19	14.4 ± 0.5
PTE	200	40.8 ± 2.1^a^	1.51 ± 0.07^a^	1.21 ± 0.43^a^	0.45 ± 0.12^a^	75 ± 8^a^	9.4 ± 0.9^b^>

N=6, Mean ± SE.

a *p*<0.05;

b *p*<0.01.

**Figure 4:**
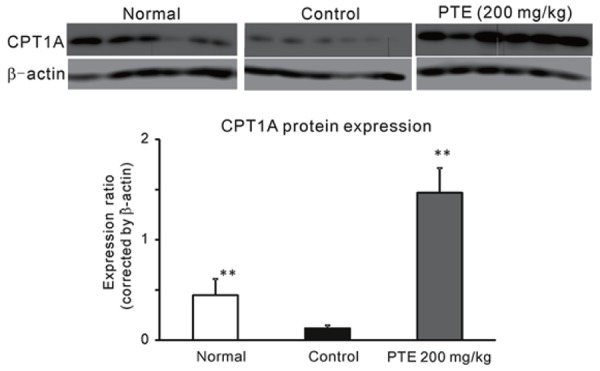
Effect of PTE on CPT1A Protein Expression in Mice Fed HFD. Each column represents mean with the S.E. (n=6). Asterisks denote significant differences from control at ^**^: *p*<0.01. PTE (200 mg/kg) was given once a day for 17 days.

Since liver weight and hepatic TG showed lower values compared to control, protein expression of CPT1A was evaluated. As shown in Fig. 4, PTE obviously enhanced CPT1A expression compared to control and normal mice.

### Effects of PTE and Its Constituents on Lipid Absorption in Mice

**Figure 5:**
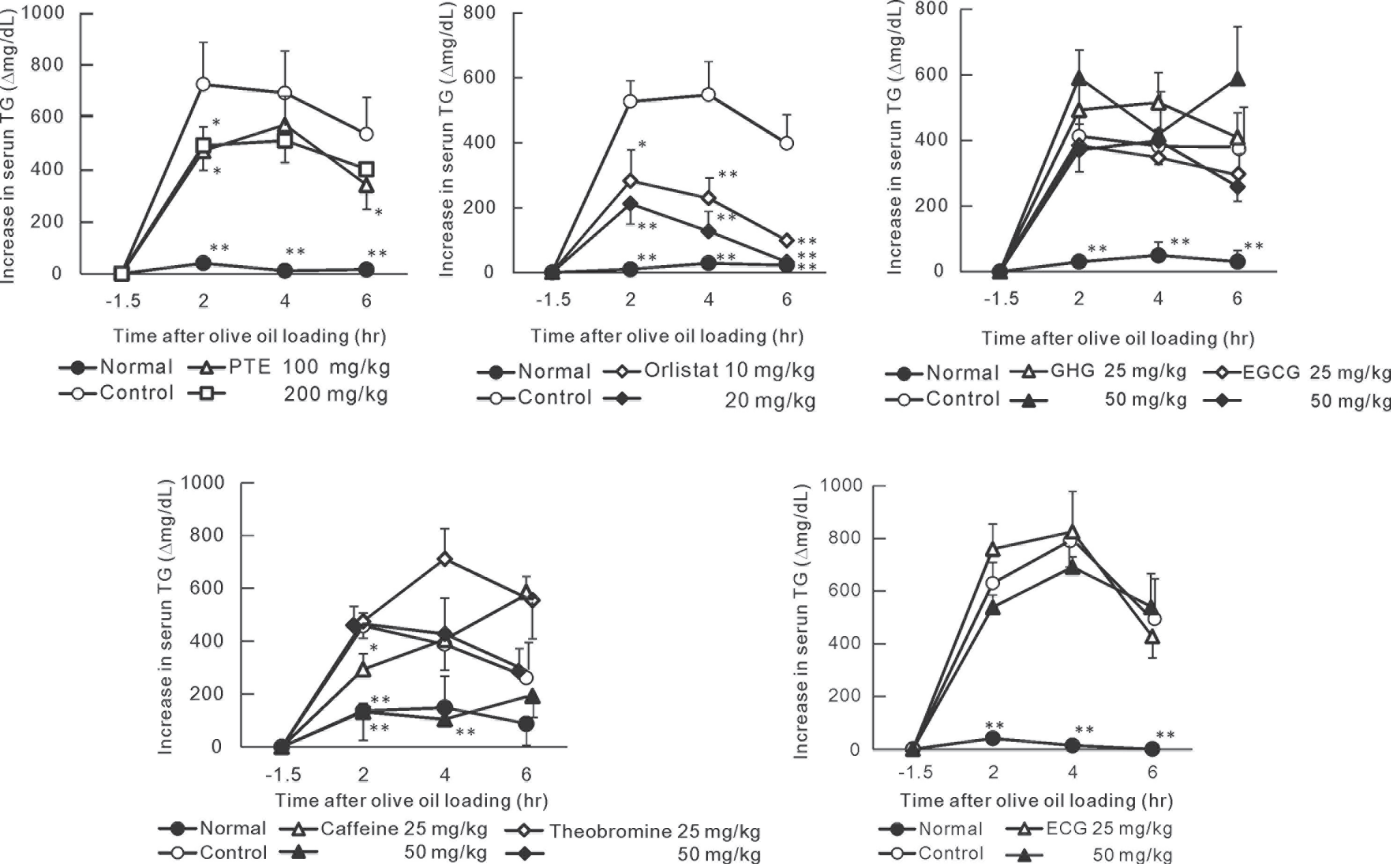
Effect of PTE and Its Constituents on Serum TG Elevation in Olive Oil-loaded Mice. Each point represents mean with the S.E. (n=6). Asterisks denote significant differences from control at ^*^ *p*<0.05, ^**^ *p*<0.01, respectively. Fasted mice were orally given PTE or its constituents and olive oil (5 mL/kg) was given 1 hr thereafter. Blood samples were collected 30 min before sample loading and 2, 4 and 6 hr later of olive oil loading.

To confirm the effect of PTE on lipid absorption from the intestines, we examined the increase in serum TG after olive oil loading. PTE (100 and 200 mg/kg) significantly suppressed serum TG at 2 hr after loading of olive oil (Fig. 5). Orlistat (10 and 20 mg/kg) used as positive control also significantly suppressed TG elevation. Among the constituents in PTE, the polyphenolic compounds including GHG, EGCG and ECG did not suppress TG elevation. Although theobromine did not suppress TG elevation, caffeine (25 and 50 mg/kg) significantly suppressed the elevation at the point 2 hrs after olive oil ingestion.

### Effects of PTE and Its Constituents on CPT1A Protein Expression in HepG2 cells

**Figure 6:**
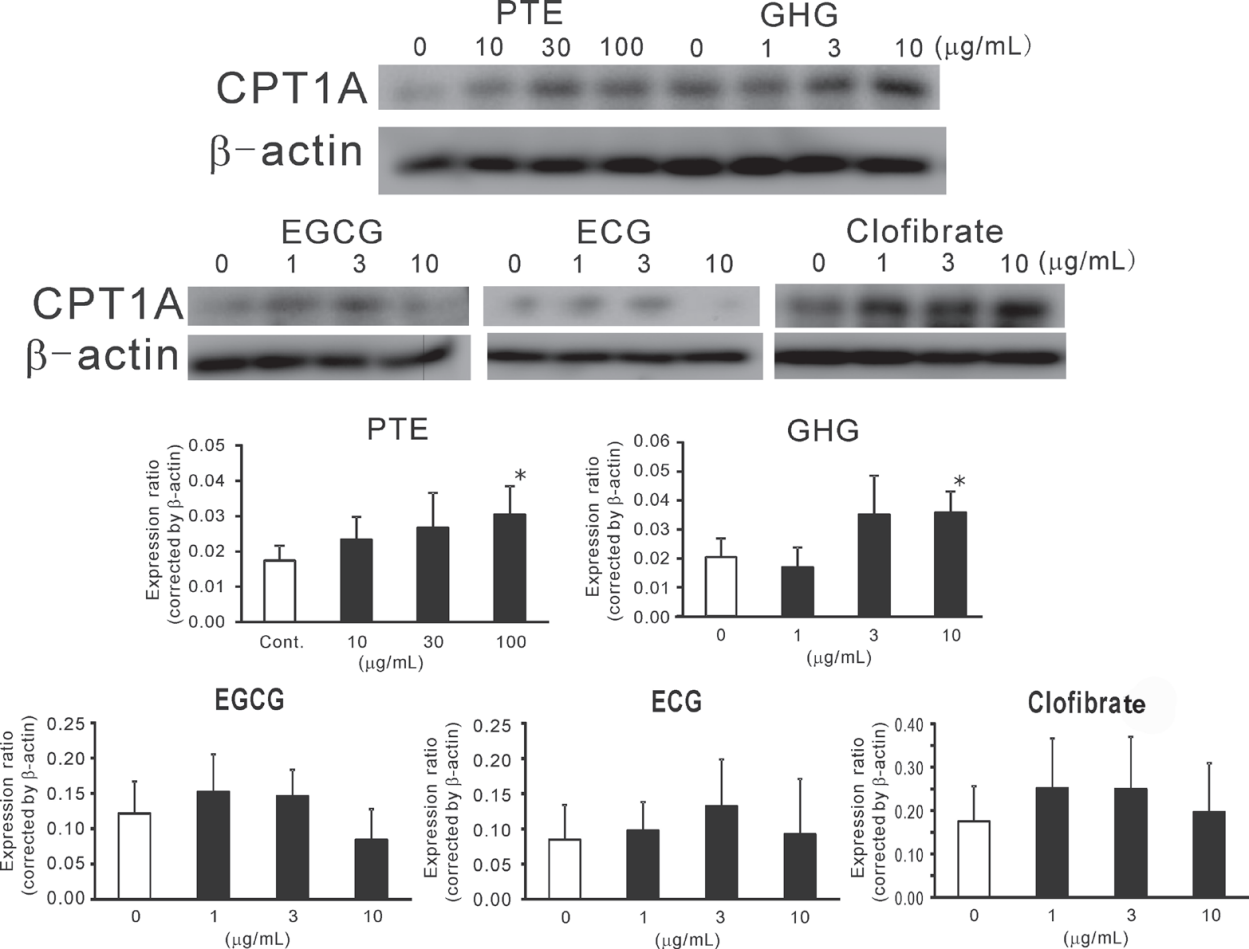
Effect of PTE and Its Constituents on CPT1A Expression in HepG2 Hepatoma. Each column represents mean with the S.E. (n=4). Asterisks denote significant differences from control at ^*^ *p*<0.05. PTE or its constituent were treated to HepG2 for 24 hr.

HepG2 hepatic cells were cultured with PTE and its constituents to examine the effects on CPT1A expression. PTE enhanced CPT1A expression from 10 to 100 μg/mL (Fig. 6). The enhancement at 100 μg/mL was significant (*p*<0.05). GHG also enhanced CPT1A expression at 10 μg/mL. EGCG, ECG and clofibrate tended to enhance CPT1A expression at 1 and 3 μg/mL, however these were not significant.

### Effect of Purple Tea on Obesity Parameters in Human

No adverse effects were observed during the test period. As shown in Table 2, after a four-week ingestion of purple tea, BMI, bodyweight, body fat mass, abdominal fat, body fat ratio, waist size, hip size, and abdominal and right upper arm fat thickness were significantly reduced compared to the values before ingestion. Muscle ratio significantly increased after the ingestion of purple tea. While there were no significant changes in blood parameters, HDL-cholesterol and HbA1c levels tended to be lower than those of before ingestion (Table 3).

**Table 2:** Comparison of Obesity Parameters before and after Four-Week Ingestion of Purple Tea

	Before Ingestion	After Ingestion

BMI (kg/m^2^)	27.0 ± 0.6	26.8 ± 0.6^a^
Body weight (kg)	80.8 ± 3.2	79.9 ± 3.1^a^
Body fat mass (kg)	21.8 ± 1.5	21.0 ± 1.4^b^
Abdominal fat (arbitral unit)	135.0 ± 8.5	123.5 ± 8.5^b^
Muscle amount (%)	24.9 ± 1.0	25.0 ± 1.0
Body fat ratio (%)	26.8 ± 1.2	26.1 ± 1.2^a^
Muscle ratio (%)	30.9 ± 0.5	31.4 ± 0.5^b^
Basal metabolism (kcal)	1789 ± 73	1768 ± 70^a^
Moisture (%)	43.2 ± 1.6	39.4 ± 4.3
Waist (cm)	97.6 ± 1.6	94.2 ± 1.7^b^
Hip (cm)	106.0 ± 1.5	102.8 ± 1.6^a^
Waist/hip ratio	0.92 ± 0.01	0.92 ± 0.01
Abdominal subcutaneous fat thickness (mm)	28.5 ± 1.4	24.4 ± 1.8^a^
Right upper arm subcutaneous fat thickness (mm)	28.5 ± 2.6	21.5 ± 1.6^b^

Values are indicated in average value and standard error (n=10). Paired *t*-test was used for evaluation of significance. Significant differences are indicated as

a *p*<0.05,

b *p*< 0.01, vs. before ingestion.

**Table 3:** Comparison of Blood Parameters Before and After Four-Week Ingestion of Purple Tea Extract

	Before Ingestion	After Ingestion

Triglyceride (mg/dL)	143.7 ± 19.4	125.9 ± 23.2
Free fatty acid (mEq/L)	0.63 ± 0.05	0.55 ± 0.04
Total cholesterol (mg/dL)	195.8 ± 8.6	183.2 ± 6.6
LDL-cholesterol (mg/dL)	120.8 ± 8.9	110.4 ± 6.8
HDL-cholesterol (mg/dL)	53.6 ± 4.7	51.9 ± 4.3^a^
Blood glucose (mg/dL)	102.7 ± 5.6	99.2 ± 4.5
HbA1c (%)	5.8 ± 0.2	5.6 ± 0.2^b^

Values are indicated in average value and standard error (n=9). Paired *t*-test among was used for evaluation of significance. No significance was observed.

a *p*=0.09;

b *p*=0.07.

## DISCUSSION

As an investigation of the anti-obesity activity of PTE, the effects on diet-induced bodyweight gain and fat accumulation were evaluated in mice. PTE (200 mg/kg) significantly suppressed bodyweight gain and abdominal and liver fat accumulations. At the same time, PTE enhanced CPT1A expression in mouse liver. In an *in vitro* assay, PTE and GHG enhanced CPT1A expression in HepG2 cells. Previously, green tea polyphenols have been reported to enhance CPT1 expression in diet-induced obesity models (19-21). EGCG, a major catechin in green tea, was hypothesized as the constituent that enhances CPT1 activity (22, 23). However, in some *in vitro* (24) or *in vivo* (25) conditions, EGCG had no influence on CPT1 expression. Sugiura *et al*. reported that combination of EGCG and caffeine enhances CPTs expression (25). Therefore, in our experiment, we investigated whether caffeine and EGCG would enhance CPT1A expression in mice fed high fat diets. In our *in vitro* experiments using HepG2, EGCG did not enhance CPT1A expression in cultured hepatoma. In fact, the effect of EGCG on CPT1A expression was ambiguous. However, GHG was shown to enhance CPT1A expression in HepG2 instead. No previous research has published the activity of GHG on CPT expression. However, structurally similar hydrolysable tannins from walnuts, tellimagrandins I and II, have been found to enhance CPT1A expression in HepG2 (17). Hence, although the combination of caffeine and EGCG might contribute to CPT1A expression of PTE in HFD-fed mice, our results suggest that GHG may also be involved in the enhancement of CPT1A expression.

Regarding intestinal fat absorption, caffeine contributed to the inhibitory effect of PTE. We have confirmed previously that caffeine contributes to an inhibitory effect of green coffee bean extract on fat absorption (18) possibly because caffeine is known to delay gastric emptying (26). Furthermore, results of previous research by Wang *et al*. indicated that caffeine significantly decreases the rate of mesenteric lymph flow, which is the route taken by absorbed fatty acids on the way to the blood stream. EGCG had no effect on lymph flow, suggesting that the effects of EGCG and caffeine on lipid absorption are mediated via distinctly different mechanisms and vary depending on types of lipids (27). The effects of caffeine are thought to contribute to reducing effect of PTE on fat absorption.

In previous clinical studies, both caffeine and catechins were reported to contribute to the suppressive effect of green tea on bodyweight gain (5). The anti-obesity effect of green tea catechins is mild and required higher dosage (800 to1200 mg/day) when they were used alone (28, 29). In fact, EGCG (300 or 1500 mg/day) has been reported to have no effect on fat accumulation in obese subjects (30, 31). Approximately 400 mg/day of green tea extract containing catechins and caffeine in combination appear to be required to exhibit anti-obesity effect (32, 33). In our study, ingestion of 3 g/day of purple tea leaves over four weeks improved obese parameters. The total amount of leaves (3.0 g/day) yielded approximately 600 mg of PTE, an extraction yield of 20%. Thus, it is reasonable in this dosage to suggest that the main ingredients (caffeine and catechins) provide the anti-obesity effects. In addition, GHG which is found uniquely in purple tea leaves at approximately the same concentration as EGCG, may also contribute to the anti-obesity effect.

In conclusion, our results indicate that purple tea leaves and its extract provide anti-obesity effects in mouse and humans and the mechanisms of action appear to utilize three compounds. Caffeine suppresses fat absorption while the combination of caffeine and catechins results in an enhanced anti-obesity effect. Lastly, GHG also may be involved in the hepatic lipid metabolizing effect of purple tea by enhancing CPT1A expression.
